# Incidence of injury and pain in referees in German national handball leagues: a cohort study

**DOI:** 10.1186/s13102-021-00320-1

**Published:** 2021-08-12

**Authors:** Jens Heyn, Johannes Fleckenstein

**Affiliations:** 1 Department of Anesthesiology, University of Munich (LMU), Marchioninistrasse 15, 81377 Munich, Germany; 2 Department of Sports Medicine, Goethe-University Frankfurt, Ginnheimer Landstr. 39, 60487 Frankfurt am Main, Germany

**Keywords:** Pain management, Sports medicine, Team sports, Play sports, Epidemiology, Biopsychosocial

## Abstract

**Background:**

Handball referees play an important role during a handball match. Surprisingly, not much is known about their sports-related injuries and resulting pain, therefore the purpose of our study was to focus on injuries and sports-related pain in referees in German handball leagues.

**Methods:**

During the 2018/19 national German handball season, referees of the German Federation of Handball (DHB) were contacted and asked to complete an injury and pain questionnaire on the penultimate matchday of the first and the second round of the season.

**Results:**

Seventy referees participated in the study. One in three referees reported an injury during the last year and perceived some form of pain. Of those suffering from pain, 16.7% referees reported chronic pain disorders. During the season, 31.4% of referees incurred an injury and the majority of the 70 referees officiated despite pain (n = 43). Prospectively-enrolled data suggested an incidence of 11.6 (95% CI: 10.3 to 13.0) injuries per 1000 match hours, and 19.0 (95% CI: 16.8 to 21.3) sports-related pain events per 1000 match hours. The most common injuries were foot and knee injuries and a substantial number of the referees (n = 25) reported taking analgesics for the pain.

**Conclusion:**

German handball referees are at risk of sports-related injuries with subsequent pain. Considering the injury profile, the incidence of sports-related pain events, and the high physiological demands of refereeing, it appears that prevention programs should be developed and integrated into the routine of the referee.

**Supplementary Information:**

The online version contains supplementary material available at 10.1186/s13102-021-00320-1.

## Background

Handball is one of the most popular sports in Europe besides football, volleyball, and basketball [1]. Since handball is a sport with high physical intensity and frequent body contact, it is not surprising that the incidence of injury is comparatively high in players [1]. Most of these injuries affect the head/neck, upper extremities including the shoulder and knee and ankle [1, 2].

Referees play an important part in handball matches. They have to be very close to the current state of play in order to get the best perspective [3]. This may result in body contact with players and in physical fatigue and consequently in an increased risk for injuries and false decision making in some cases [3]. The fact that referees are often older than players may exacerbate these risks and consequently, referees in modern handball should be trained adequately [3, 4].

Although referees are at risk of injuries during handball matches, research dealing with this issue and possible consequential damages and symptoms is scarce. Especially, localization of the injury, restrictions due to the injury, and pain levels have not been well analyzed. Therefore, the aim of our study was to (i) analyze the incidence of injuries in handball referees, (ii) to examine localization of the injury and its underlying mechanism, (iii) to explore the consequent restrictions, and finally (iv) to evaluate referees’ pain levels.

## Methods

### Study design

**Fig. 1:**
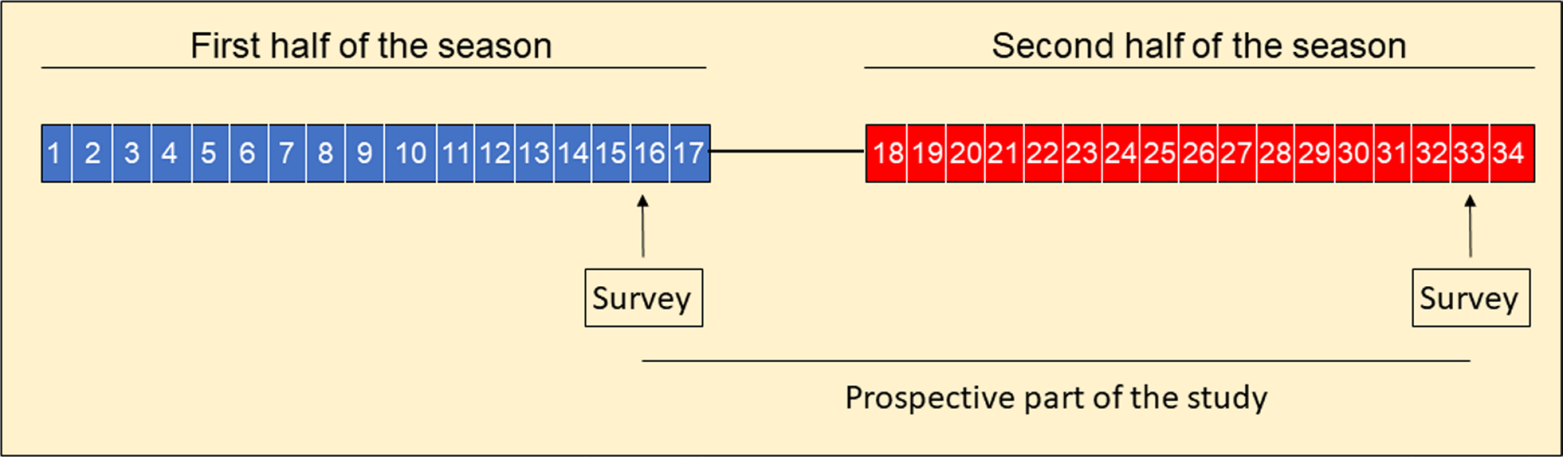
Study design. The survey was carried out on the penultimate matchday of the first round and the penultimate matchday of the second round of the first 1. German Handball League. The time between the two survey times represents the prospective data collection. All referees leading matches in the 1.-3. German Handball League and the 1. German Women Handball League were asked to participate in the study. Numbers represent the respective matchday. Although the leagues differed in the number of match days (from 26 match days in the 1. German Women Handball League to 38 matchdays in the 2. German Handball League), their season schedule was relatively similar. The full season of the different leagues lasted from August/September 2018 to May/June 2019

We conducted an exploratory paper-based cohort study assessing pain and injury among referees in the German handball leagues during the season 2018/19. Questioning was conducted during the penultimate matchday of the first round of the season. For prospectively-enrolled data, referees were encouraged to participate in the survey again on the penultimate matchday of the second round of the season (see Fig. 1). Still, all data collected by means of questionnaires is retrospective by its nature.

### Participants

Potential participants were all referees registered with the German Federation of Handball (DHB) at the time of the survey. Referees who did not participate in the 1.-3. German Handball League (men) or in the 1. German Women Handball League and referees who retried during the season were excluded. The questionnaires were distributed by the Referee Chairman of the DHB, and anonymized completed questionnaires were sent to us by those referees who gave written informed consent. In total, 270 referees received the survey and seventy of these referees completed the survey.

### Outcome measures

The questionnaire comprised of the following questions (see Table 1):

**Table 1:** Composition of the questionnaire

Demographics	Age, sex, height, weight	
	Experience being a referee	
	Training loads	Endurance, strength, circuit
	Occupation	
	Number of actions	Per week, last 4 weeks
	International participations	
Injury	1-Year Incidence of sports-related injury	Match, training
	Incidence of injury during season	
	Type of injury	
	Mechanism of injury	
	Degree of disability	
	Therapy	
Pain	Sports-related pain	Occurrence, location, intensity
	Use of analgesics	Type, frequency

The complete questionnaire is accessible as Additional file 1: File S1.

### Definitions

Physical overload: Overload basically means that an exercise must become more challenging during a training program for the purpose to increase fitness and robustness. However, in some cases the overload is outside a healthy level and results in injury and or harm. Physical overload should be interpreted in this context when exploring the results of this study.

NSAID: Nonsteroidal anti-inflammatory drugs are medications mostly prescribed for the treatment of painful conditions. Most people are familiar with over the counter, non-prescription NSAIDs. Besides its pain relief effect, NSAIDs also have anti-inflammatory properties and reduce swelling.

NRS: The Numeric Rating Scale (NRS) is a simple and often used numeric scale in which people rate their pain from 0 (no pain) to 10 (worst pain).

Sports-related injuries: All injuries related to the activities related to sport activities (including training).

Point-prevalence: Prevalence of a disorder at a specific point in time.

### Statistical analysis

Statistical data processing was performed using SPSS (Version 24; IBM Deutschland GmbH, Ehningen). Since all data had a normal distribution (Kolmogoroff–Smirnoff–Lilliefors test), data are expressed as means and standard deviations (95% CI). For statistical analysis between different subgroups, the T-test for independent samples and the Chi^2^ test for categorized data were applied. The level of statistical significance was set at *p* < 0.05. Correlations were calculated applying Pearson’s coefficient, and are indicated as Pearson’s r.

## Results

### Characteristics of the referees

Seventy referees participated in the study—their characteristics are summarized in Table 2. It should be noted that the average BMI of female referees was approximately 2 kg/m^2^ lower than the average BMI of male referees. The training frequency of the referees ranged between two and five days with an average of three days.

**Table 2:** Characteristics of referees who are suffering from an injury and of those who did not. Data presented as mean ± standard deviation

		Referees with injury	Referees without injury
Demographics	Age [years]	31.3 ± 7.8	32.0 ± 6.8
	Female gender [n]	4	3
	BMI [kg/m^2^]	24.8 ± 2.7	24.9 ± 1.8
Training habits	Daily training time [h]	1.5 ± 0.9	1.1 ± 0.7*
	Endurance training [%]	61.9 ± 22.5	66.1 ± 24.9
	Strength training [%]	29.2 ± 21.1	24.8 ± 21.4
	Circuit training [%]	6.1 ± 10.8	5.8 ± 10.9
Referees' characteristics	Experience [years]	15.7 ± 6.5	15.7 ± 6.4
	Commitment international [%]	14	20
	Commitment 1. league [%]	23	32
	Commitment 2. league [%]	40	56
	Commitment 3. league [%]	43	60
	Commitment in 1. league women [%]	37	52
	Match load [hours/week]	1.1 ± 0.4	1.0 ± 0.3

*p < 0.05

### Retrospectively reported injuries

Twenty-two (31.4%) referees had suffered a sports-related injury within the last year. Most of the injuries occurred during training. In total, 28 injuries were reported, with foot (53.6%) and knee (32.1%) injuries being most common (Table 3). Injury mechanism included turns and changes of direction (29.7%), as well as sprints (21.6%) and physical contact with players or ball (21.6%). Physical overload was reported by none of the referees. Due to their injury, daily living activities were affected: refereeing (72.0%), walking (60.0%), stair climbing (44.0%), occupation (24.0%), car driving (20.0%), and sleeping (16.0%). Therapy included physiotherapy (64.0%), analgesics (44.0%), taping (40.0%), massage (24.0%), trans-dermal electro stimulation (12.0%), and surgery (12.0%).

**Table 3:** Injuries reported by referees during the last year. Absolute numbers are given in the table. The reported injuries included the calendar year 2018

	Match	Training	Both	Total
Foot	1	12	2	15
Knee	1	6	2	9
Pelvis	0	1	1	2
Chest	0	1	0	1
Hand	0	1	0	1
Total	2	21	5	28

### Retrospectively reported pain

Sports-related pain was reported by 24 (34.3%) referees during the last year; the pain occurred, immediately after the match in 37.5% of the (9/24 referees), on the day after the match (9/24; 37.5%), up to 4 days (3/24; 12.5%), up to one week (1/24; 3.1%), or continuously (3/24; 12.5%). The mean pain intensity perceived among referees was 3.0 ± 1.8 Numeric Rating Scale (NRS) with a maximum perceived pain of 5.3 ± 2.2 NRS and a minimum pain of 0.9 ± 1.1 NRS. The point-prevalence of pain intensity at the time of the survey was 1.6 ± 1.8 NRS. Fifteen (21.4%) referees reported using analgesics with different frequency.

Twenty-six (37.1%) referees reported that they officiated at matches despite suffering from pain, and 8 (11.4%) referees reporting doing so regularly.

### Other retrospective information

To gain a better understanding of causes for injuries, we divided the referees into two groups. Group 1 included all referees who suffered an injury within the last year, the second group (group 2) of referees did not have an injury during the last year. We found an association with training load and injury during the last year (trainings load per day in group 1: 1.5 ± 0.9 h vs. trainings load per day in group 2: 1.1 ± 0.7 h; *p* = 0.04). Significant more referees who experienced an injury during the last year officiated a handball match despite of pain (*p* < 0.02).

Referees suffering from sports-related pain mediated an increased mean pain intensity (NRS: 3.4 ± 1.6 vs. 1.4 ± 1.3; *p* < 0.02). Furthermore, we found some moderate correlations: age (r = 0.493; *p* < 0.02) and experience (r = 0.499; *p* < 0.02) were associated with a higher frequency to use analgesics. A higher BMI moderately correlated with increased analgesic intake (r = 0.441; *p* = 0.04), and mean pain intensity (r = 0.420; *p* < 0.04).

### Prospectively-enrolled reported injuries and pain

Fifty-two of the 70 referees (74.3%) responded in the prospectively-enrolled part of the survey. Seventeen (32.7%) referees suffered a sports-related injury during the observational period, with injuries occurring more often during training than during a match. In total, 27 injuries were reported, with foot (51.9%) and knee (25.9%) injuries being most common (Table 4). Injury mechanism included turns and changes of direction, as well as sprints (both 27.6%). Physical overload was reported by four of the referees. Due to their injury, daily living activities were affected: refereeing (82.4%), walking (82.4%), stair climbing (58.9%), car driving (35.3%), occupation (29.4%), and sleeping (11.8%). Therapy included physiotherapy (82.4%), analgesics (64.7%), taping (64.7%), and massage (35.3%). A physician attended 41.2% of the injured referees.

**Table 4:** Injuries reported by referees during the season 2018/19. Injuries are stated as absolute numbers

	Match	Training	Both	Total
Foot	5	8	1	14
Knee	3	1	3	7
Pelvis	0	1	1	2
Trunk	0	1	0	1
Arm	0	1	0	1
Shoulder	0	1	0	1
Head	0	1	0	1
Total	8	14	5	27

The cumulative training load of 205 h per week, resulted in 2.6 injuries per 1000 training hours (95% CI: 2.09 to 3.31). Taken cumulatively, 56 officiated matches per week resulted in 11.6 injuries per 1000 match hours (95% CI: 10.3 to 13.0).

Most referees reported officiating despite having pain (n = 43). 18 referees reported sports-related pain during the season. Their mean pain intensity during the season was 2.3 ± 1.4 (maximum pain 4.8 ± 2.7; minimum pain 1.0 ± 1.4). The point-prevalence of pain intensity at the time of the survey was 1.5 ± 1.8, and pain intensity immediately after their last officiated match was reported to be 3.1 ± 2.2. 20 (46.5%) of these pain-affected referees reported the use of analgesics. Furthermore, 67.4% of these referees also agreed to the sentence that a referee need to be ready to tolerate pain due to sporting reasons. The accordance with this statement was significantly higher in referees suffering from pain than in those without pain (*p* < 0.04). The incidence of sports-related pain was 19.0 (95%-CI 16.8 to 21.3) per 1000 match hours.

### Gender differences

Although the number of female referees was low in our study, we analyzed if there are any gender specific differences between female and male referees. We run this sub-group analyses with the knowledge that the statistical power might be insufficient to have robust results. However, aside anthropometric data (height, weight, BMI), we could not detect any other differences when comparing female and male referees.

## Discussion

To our knowledge, this is the first specific report on pain being a severe symptom among referees in sports. Whereas previous surveys focus on injuries and functional complaints, this recent research emphasizes the need for remedies to prevent and relieve pain. Based on a sample of national handball referees our data suggest one out of three referees suffer from pain, one out of three officiate despite pain, and almost every tenth to do so regularly. The mean pain intensity in all referees was three, independently of the time of surveying. Given a mean pain intensity of three is alarming, as this pain level is classified as light to moderate and is assigned to Step 1 of the pain ladder according to the standard WHO Pain Management guidelines [5]. Consequently, considerable number of the referees surveyed would need pain therapy. Step 1 implies the need for at least non-opioid analgesics, in particular nonsteroidal anti-inflammatory drugs (NSAID), for pain therapy.

Our data reporting injuries and injury rates (1-year prevalence 36%; season prevalence 32.7%) are similar to injury rates for referees in other types of sports. In Iran’s Football Premier League, 6-month injury incidence was reported to be 22.4%, 31 out of 59 (56%) referees reported a history of knee injury, and injured referees reported decreased activities in daily and recreational living [4]. In Gaelic football, the annual injury prevalence was 58%, with 14% injured at the time of the survey [6]. Almost every third injury led to time off from refereeing for a median duration of 3 weeks and this study reported that more injuries occurred during matches (60%) than during training. Data on Swiss Football League referees suggested that injuries were more frequent during training than during matches [7]. Our data suggests that injuries were more likely to occur following matches than training when taking into account the number of match / training hours. However, since the training load equals approximately 7-times the weekly match load, the probability of suffering injury in training also increases. Based on our results, the duration of the training seems to play an important role. Referees who had an injury had a 0.4 h longer daily training duration than their colleagues without an injury. These 0.4 h seems to be not clinically meaningful at first glance, still it is a 36% increase in training load. Taking into account that handball referees have a usual occupation (with 40 h at work per week) and the training is additionally, this moderate difference could be important. Increased training load required additional recovery [20]. Disturbed recovery as well as overtraining may result in long-term decrement in performance capacity with or without related physiological and psychological signs and symptoms. In brief, this may present a substantial risk of injury and pain.

Bizzini et al. confirmed their previous results [7] in a survey of the soccer referees at the 2006 FIFA world cup [8]. They demonstrated that 33.3% of the referees suffered from low back pain. However, reporting on pain focused on musculoskeletal disorders and did not specify the impact of pain on injury and daily living. Other studies dealing with pain or pain-like symptoms are sparse. In a study investigating the prevention on muscle soreness in soccer referees by means of absorbing heel inserts, it was hypothesized that 80% of referees suffered from muscle soreness [9]. One should be aware, that muscle soreness is a symptom complex including in particular pain, stiffness and weakness in the affected area others, and is more likely to be an acute and subacute disorder than an equivalent to chronic pain [10].

Previous reports about injuries in soccer referees indicated that these referees resumed refereeing as early as one week after the injury occurred [11]. In some cases, the referees restarted with referring before the injury was cured or before injury related symptoms disappeared [4, 7, 11]. This insufficient recovery might increase the risk of a re-injury and therefore endangers referees’ health and well-being [4, 11]. It is also known that the referees’ health and quality of refereeing are directly related [4, 11]. These findings in soccer referees are comparable to handball referees. Handball referees also tend to resume refereeing early with a substantial portion of them still having pain and or symptoms. Soccer and handball referees make use of the medical system or of treatment option after an injury occurred in a similar way. Both groups of referees most frequently use physiotherapy [4].

Current literature provides evidence that BMI and distribution of body fat influence sensory detection and pain sensitivity [12]. Obesity was linked to an increased sensitivity of pain. This phenomenon can be also found in patients after operation. Juhl et al. [13] showed a significant correlation of pain after mastectomy with the BMI; women with an increased BMI had higher pain levels. Furthermore, a connection of BMI and the use of pain killers has been evaluated in the past. Lendrum et al. [14] showed that higher BMI is associated with increased opioid consumption after surgery. These findings in patients are also applicable to handball referees. In handball referees we found a positive correlation of the BMI with pain levels and the frequency of pain killer usage.

Besides injury and pain, it is the psychological demands on referees that could play an eminent contributing role [8]. We did not assess the occurrence of biopsychosocial factors within this survey, such as e.g. depression, anxiety, stress, self-compassion, alcohol consumption, as well as sleep quality, health-related quality of life, or impairments of quality of life. However, related data from studies on pain among sports students suggest the high relevance of social and supogical factors contributing to chronic pain and injury in sports [15]. There is one large study investigating the sources of stress among Greek handball referees [16], which ranked the following stressor factors highest: "Making a wrong call", "Lack of cooperation with my partner", and "Refereeing an important game". Environmental discomfort has been proposed another important factor [17]. Referees in professional football, were surveyed for common mental disorders, and a one-season incidence of 10% for distress, 16% for anxiety/depression, 14% for sleep disturbance, 29% for eating disorders, and 8% for adverse alcohol use was reported [18]. Finally, self-efficacy has been proposed as being another important contributing factor to the occurrence of pain [15]. It has been shown that years of experience and physical/mental preparation are predictive of all factors of referee self-efficacy [17]. In our study, we could show that referees’ experience predicted analgesic consumption. A link between analgesics and self-efficacy remains hypothetical, but seems likely. Altogether, evaluation of these co-factors may be helpful for further research and should be subject to further studies.

## Limitations

We are aware that our data is based on a voluntary survey, distributed among referees with the help of the German Federation of Handball (DHB). The ratio of supposed non-responders is in line with similar research, but we cannot rule out that their non-response may have altered the results. Furthermore, the number of female referees in our study and in general is low. We can therefore not rule out that analyzing female referees (only) might reveal other results. Additionally, we did not assess if the referees are performing any other sport or if the referees had a professional career as a handball player before starting their career as a referee. It might be possible that these factors might have an influence on our results. The assessment of pain and injury is based on simple dichotomous or categorized questions, and linear data was acquired using numeric rating scales. This is the simplest way to get an overview about a topic that hitherto had been addressed only in parts. A more comprehensive assessment applying a battery of validated questionnaires, as recently promoted [15], would have revealed a more detailed description. We decided to use a very shortened and reduced survey, as we could not estimate whether referees were willing to respond or not. As handball referees present a small cohort, our primary aim was to obtain as many responders as possible. Reporting and interpretation of the results is based on the guidelines for of the STROBE initiative [19]. We assume our sample size to be adequately robust to represent the chosen cohort. Finally, the comparison of pain experienced by handball referees to referees in other disciplines is limited. Altogether, data reporting on referees is very limited and conclusions must be drawn carefully.

## Outlook

Best injury and pain prevention could start which an adequate education of referees (athletes and staff). A profound risk awareness as well as the knowledge of red and yellow flags is essential to assess the extent of the own training. As stated above, training loads must be compensated by adequate recovery, and in case of excessive loads by adequate rest [20]. This must be ensured, and affected people must be strongly encouraged to follow recovery plans. Finally, the real training program is individually designed, it should be based on regularly performed cardio-pulmonary exercise testing, and include a balanced mix of endurance, resistance and flexibility exercises with variable loads.

## Conclusion

This survey shows that referees in German national handball leagues are at considerable risk for sport related injuries resulting in pain. In some cases, referees complained about signs of chronification, and there are hints that other biopsychosocial cofactors may play an important contributing role.

Erickson and colleagues published a weighty statement in 2012, that “umpires, referees, and sports officials should deserve the same attention by the sports medicine community as the athletes with whom they share the field” [6]. Summarizing the findings of our survey, we would like to suggest an amendment to this requirement that this attention should be directed to preventive strategies not only to prevent injuries but also to prevent the occurrence of pain disorders.

## Supplementary Information

**Additional file 1: File S1.** Questionnaire used in this study.

## Data Availability

The datasets used and/or analyzed during the current study are available from the corresponding author on reasonable request (in compliance with data privacy).

## Abbreviations

CI: Confidence interval
NRS: Numeric rating scale
BMI: Body mass index
NSAID: Nonsteroidal anti-inflammatory drugs
WHO: World Health Organization
