# Supplementary material for: Incidence of injury and pain in referees in German national handball leagues: a cohort study
Source: BMC Sports Sci Med Rehabil. 2021 Aug 12;13:88. doi: 10.1186/s13102-021-00320-1 (PMC8359112; doi:10.1186/s13102-021-00320-1)
Supplement: Supplementary file 1 — Additional file 1: File S1. Questionnaire used in this study. [file 13102_2021_320_MOESM1_ESM.docx]

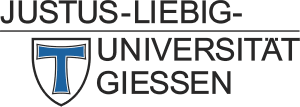

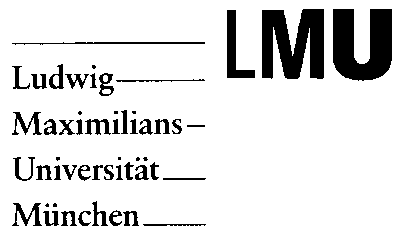


File S1. Questionnaire used in this study.

Note: The original questionnaire was designed in German. This is an English translation to make the study easier to understand. The original file can be obtained from the authors on request.

Age _______ years Gender ________

Hight ________ m Weight ________ kg

Experience as referee _______ years

Duration of training _______ hours per day, including

Endurance training __________ in %

Strength training __________ in %

Circuit training __________ in %

Professional activity:_______________________

When do you do your training?

morning afternoon evening

Do you perform your training after your main work?

never seldom often always

How many training days do you have per week? __________ days

How long is your training? ­________ hours/day

Do you perform a warm-up before starting with the training?

yes no

Do you perform stretching during the training?

yes no

Do you perform a cross-training to claim different groups of muscles and joints?

yes no

Do you have training session when you are ill?

yes no

Do you make use of periodic medical check-up in a department of sports medicine?

yes no

I am working as referee in (multiple answers possible)

league 1 league 2 league 3 women’s league 1

Average number of matches per week

< 1 1 2 > 2

Number of matches during the last 4 weeks

0-2 3-4 4-6 > 6

Participation in international matches?

yes no

Have you had an injury which was related to handball during your career?

yes no how many? ________

Have you had an injury which was related to handball during last season?

yes no

When did the injury occurred?

first half of the season second half of the season

When did the accident/injury occur?

match training

Which area was affected?

food/ankle knee

pelvis thorax

hand arm/shoulder

head

Mechanism which lead to the injury

contact with a player or sporting equipment running/sprint

rotation/change of direction overload

__________________

Impairment

as a referee during the main job

driving walking

climbing stairs sleep

Therapy

pain killer physiotherapy

TENS (transcutaneous electrical nerve stimulation) massage

tape operation

others:_________________

Do you have pain associated to sport?

yes no

If yes, when do you have the pain?

after the match the day after the match

up to 4 days after the match up to seven days after the match

always

Was the pain increasing during the season?

yes no

Where is the pain located? ____________________________________

Average pain 0---1---2---3---4---5---6---7---8---9---10

Maximum pain during last month 0---1---2---3---4---5---6---7---8---9---10

Minimum pain during last month 0---1---2---3---4---5---6---7---8---9---10

Present pain 0---1---2---3---4---5---6---7---8---9---10

[0 = no pain, 10 = maximum pain]

I take the medication ibuprofen/diclofenac

in general only in painful situation prophylactic (before match/training)

never

Do you participate in matches as referee suffering from pain?

yes no regular

If yes, do you need painkillers?

yes no

Average pain after a match 0---1---2---3---4---5---6---7---8---9---10

How do you assess the following sentence?

When I am working as a referee, you need to be ready to tolerate pain due to sporting reasons. Please mark the answers which matches best for you.

I do not agree I agree

Thank you very much for your participation!
